# Supplementary material for: Orchestrating NK and T cells via tri-specific nano-antibodies for synergistic antitumor immunity
Source: Nat Commun. 2024 Jul 23;15:6211. doi: 10.1038/s41467-024-50474-y (PMC11266419; doi:10.1038/s41467-024-50474-y)
Supplement: Supplementary file 1 — Supplementary Information [file 41467_2024_50474_MOESM1_ESM.pdf]

Supplementary Information
for

Orchestrating NK and T cells via tri-specific Nano-Antibodies for synergistic antitumor immunity

Qian-Ni Ye, Long Zhu, Jie Liang, Dong-Kun Zhao, Tai-Yu Tian, Ya-Nan Fan, Si-Yi Ye, Hua Liu, Xiao-Yi Huang, Zhi-Ting Cao, Song Shen*, Jun Wang*

* Address correspondence to and shensong@scut.edu.cn (S. Shen) and mcjwang@scut.edu.cn (J. Wang)

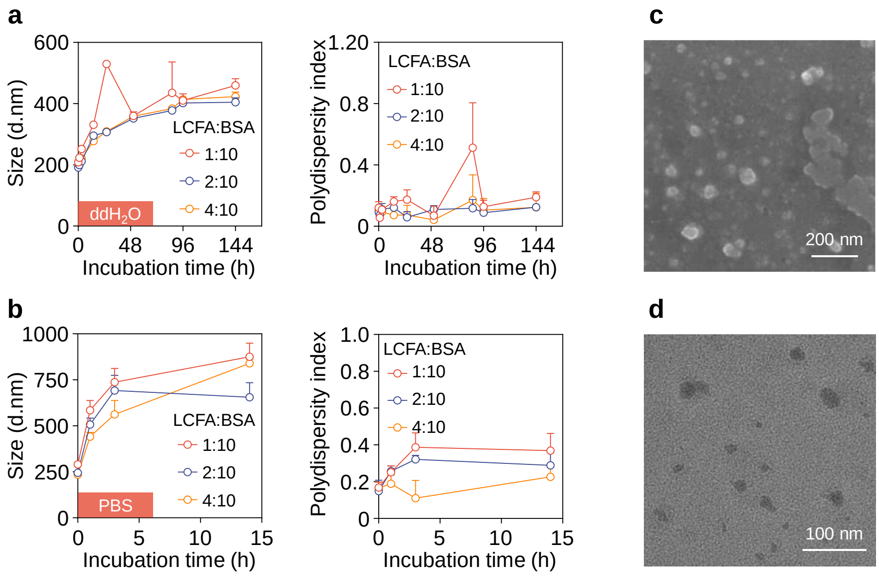

Supplementary Fig. 1. Characterization of albumin/LCFA composite nanoparticles. Variation of the size (left) and PDI (right) of albumin/LCFA (stearic acid) composite nanoparticles in ddH$_2$O (**a**) and PBS buffer (**b**). PDI, polydispersity index. LCFA, long-chain fatty acids. Representative SEM (**c**) and TEM image (**d**) of albumin/stearic acid composite nanoparticles assembled by stearic acid and BSA with a mass ratio of 4/10. BSA, bovine serum albumin. Data of size and PDI are presented as means ± s.d. (n = 5 biologically independent samples). Source data are provided as a Source Data file.

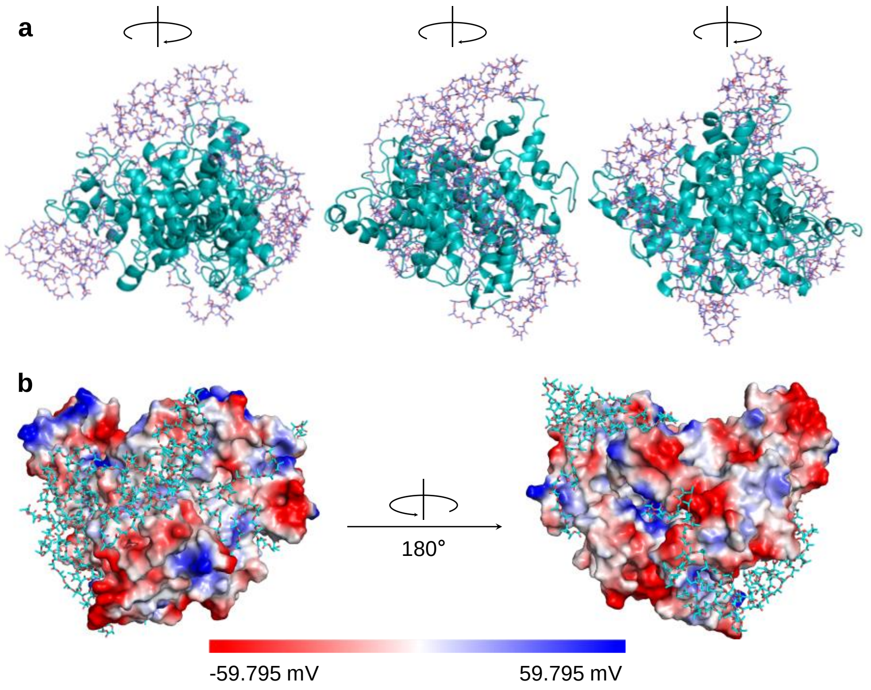

Supplementary Fig. 2. Molecular dynamics simulations. (a) The resultant molecular dynamics simulation pattern of PLLA complexed with HSA from different angles. **(b)** HSA surface potential. The PLLA used has a polymerization degree of 400 and has ester end groups (PLLA-COOR, R=-(CH_2)$_{11}$$CH_3$). HSA: human serum albumin.

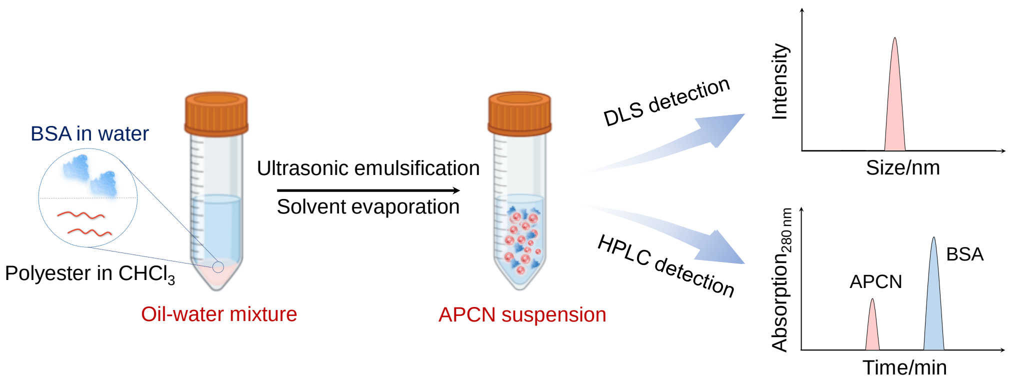

Supplementary Fig. 3. Screening process of the optimal APCN. APCN was formulated by a single emulsification-solvent evaporation method. Briefly, an aqueous solution of BSA (10 mg/mL) and LCFA or polyesters dissolved in chloroform (5 mg/mL) were ultrasonically mixed at a volume ratio of 5/1 over an ice bath using an ultrasonic processor (Sonics & Materials, Inc.). The emulsion was then concentrated to remove organic solvent by a rotary evaporator (IKA). Finally, the nanoparticle suspension was characterized by DLS and filtered using a 0.22 μm syringe filter for quantitative detection of albumin loading efficiency by HPLC (Waters, Ultrahydrogel Column 500 Å). Supplementary Fig. 3 was created with BioRender.com under a CC-BY-NC-ND license.

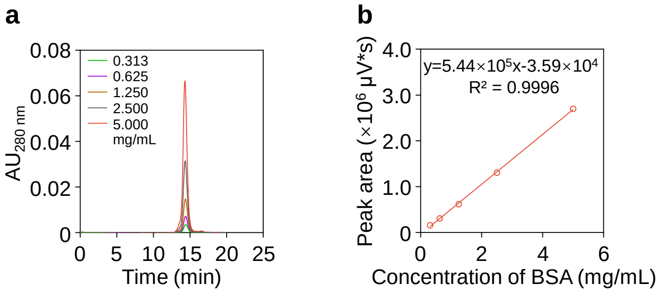

Supplementary Fig. 4. Chromatograms and standard curve of BSA examined by HPLC. (a) Representative chromatograms of BSA with different concentrations. **(b)** Linear fitting analyses of chromatogram integral area to corresponding concentrations of BSA, indicating a significant correlation (R^2=0.9996). Source data are provided as a Source Data file.

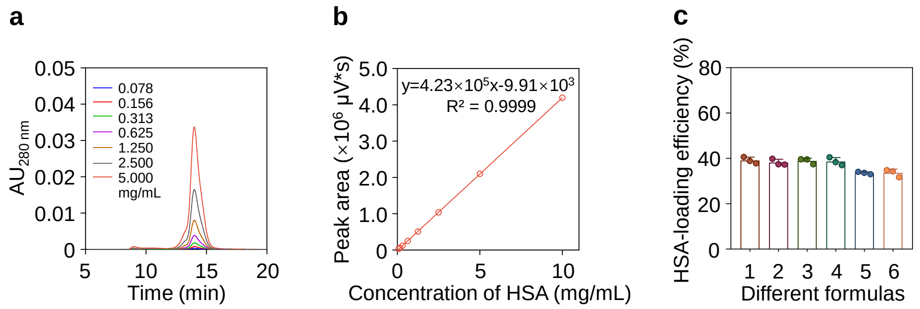

Supplementary Fig. 5. The HSA loading efficiency of APCN constructed from different formulas. (a) Representative chromatograms of HSA with different concentrations. **(b)** Linear fitting analyses of chromatogram integral area to corresponding concentrations of HSA, indicating a significant correlation (R^2=0.9999). **(c)** The HSA loading efficiency of APCN constructed from different formulas according to the fitted equation. Data are presented as means ± s.d. (n = 3 biologically independent samples). Source data are provided as a Source Data file.

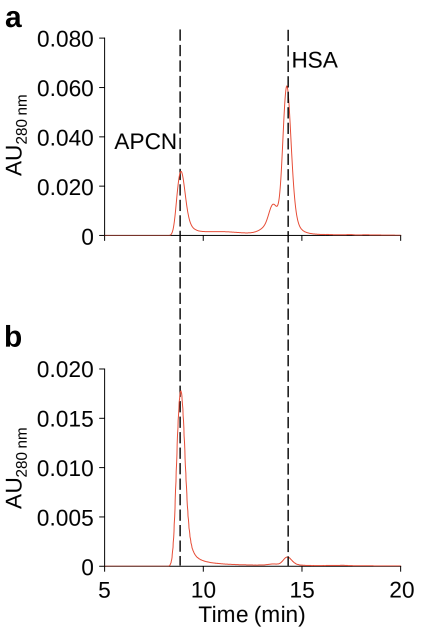

Supplementary Fig. 6. Representative chromatograms of the purified APCN. APCN was purified through high-speed centrifugation at 4°C (21000 g for 60 min). Then APCN before (**a**) and after (**b**) purification was detected by HPLC (Waters, Ultrahydrogel Column 500 Å). Source data are provided as a Source Data file.

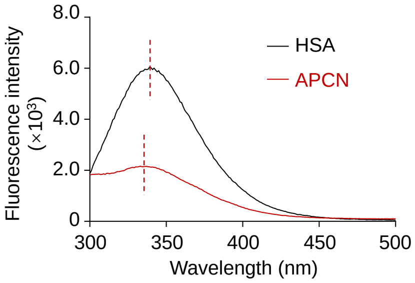

Supplementary Fig. 7. Fluorescence spectra of HSA or APCN. HSA or APCN was dispersed in ddH_2O and fluorescence spectra was scanned from 300 to 500 nm at an excitation wavelength of 280 nm using Shimadzu RF-6000 Fluorescence Spectrophotometer. Excitation and emission bandwidth were 10 nm. Source data are provided as a Source Data file.

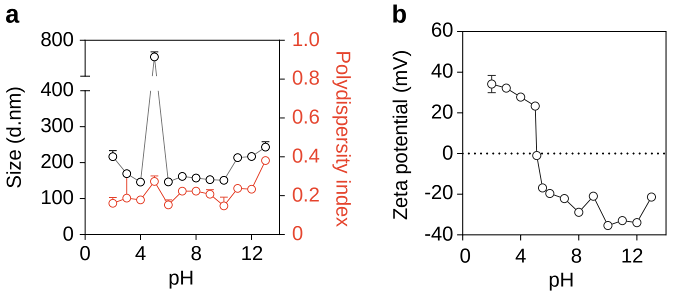

Supplementary Fig. 8. The size and zeta potential of APCN in PBS with a pH of 2-13 by DLS. Data are presented as means ± s.d. (n = 5 biologically independent samples). Source data are provided as a Source Data file.

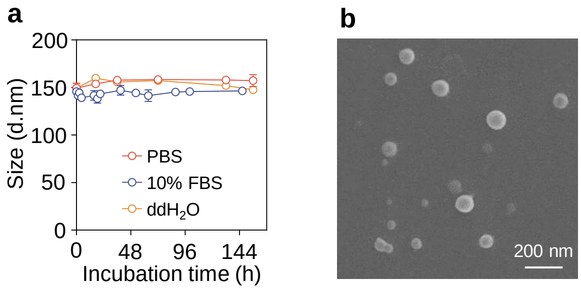

Supplementary Fig. 9. Stability of APCN in vitro. (a) The size of APCN in ddH$_2$O, PBS or PBS containing 10% FBS (v/v) after 6 days of incubation. **(b)** Representative SEM image of APCN after storage in water for a week. Data are presented as means ± s.d. (n = 5 biologically independent samples). Source data are provided as a Source Data file.

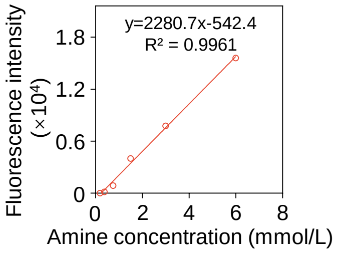

Supplementary Fig. 10. Standard curve for detection of amino density. Source data are provided as a Source Data file.

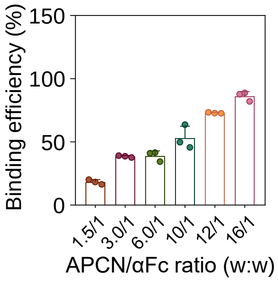

Supplementary Fig. 11. Binding efficacy of αFc. Through centrifugation, the unbound αFc in supernatants was examined by enzyme-linked immunosorbent assay (ELISA, goat IgG ELISA Kit, Alpha Diagnostic International). Data are presented as means ± s.d. (n = 3 biologically independent samples). Source data are provided as a Source Data file.

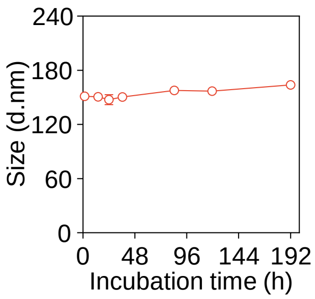

Supplementary Fig. 12. Stability of APCN@NA in ddH$_2$O after incubation for 8 days. Data are presented as means ± s.d. (n = 5 biologically independent samples). Source data are provided as a Source Data file.

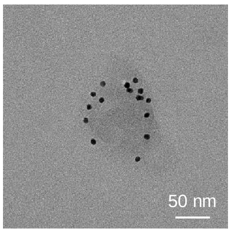

Supplementary Fig. 13. Representative immune electron microscopy image showing the binding ability of APCN@NA to antibody. To confirm that mAb could be immobilized on APCN@NA, $NP_{αPDL1}$ was prepared by gently mixing αPDL1 with APCN@NA at the αPDL1/αFc ratio of 1:1 (w/w) and incubated for 24 h at 4 °C, and then anti-Rat IgG (whole molecule)-Gold antibodies were added to $NP_{αPDL1}$ for 6 h at 4 °C. Finally, the mixture was purified via high-speed centrifugation (21000 g for 10 min) at 4°C, and the purified nanoparticle was detected by TEM.

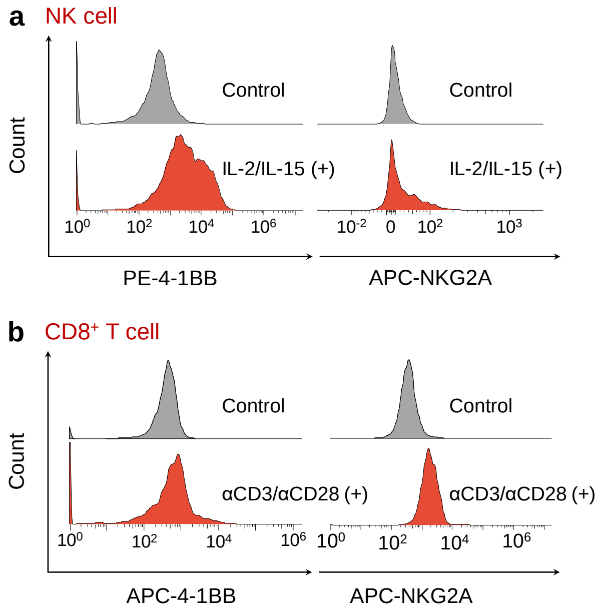

Supplementary Fig. 14. Expression levels of 4-1BB and NKG2A on stimulated NK and CD8$^+$ T cells. Spleens removed from C57BL/6 mice were placed on a steel wire mesh in pre-cooled sterile PBS to be gently fragmented and the splenocyte suspension was filtered through a 40-μm nylon mesh. Red blood cells were removed by erythrocytes lysate (Biosharp), and splenocytes were then resuspended in magnetic-activated cell sorting (MACS) buffer and NK/T cells were isolated via a mouse CD49b (DX5) and CD8a (Ly2) MicroBeads (Miltenyi Biotec). For NK cell stimulation, isolated NK cells were incubated with IL-2 (20 ng/ml, PeproTech) and IL-15 (20 ng/ml, PeproTech) for 4 days. For T cell stimulation, isolated CD8$^+$ T cells were incubated with plate-bound anti-CD3 antibodies (5 μg/mL, BioLegend) and soluble anti-CD28 antibodies (5 μg/mL, BioLegend) for 24 h. 4-1BB and NKG2A expressions was assessed using flow cytometer (BD Biosciences). The data were analyzed using FlowJo software (version 10.0.7, TreeStar Inc, Ashland, USA).

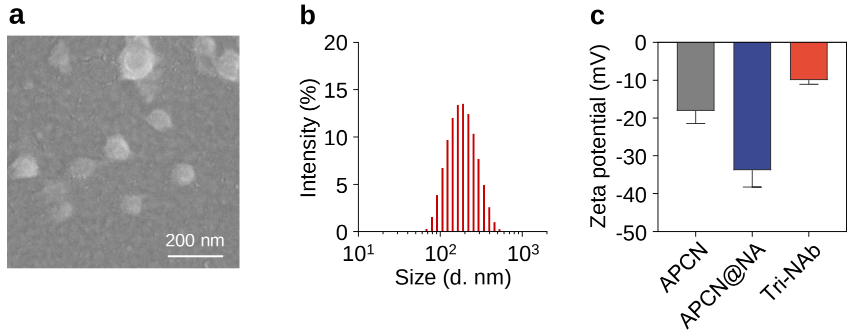

Supplementary Fig. 15. Characterization of Tri-NAb. (a) Representative SEM image of Tri-NAb. **(b)** Size distribution of Tri-NAb. **(c)** Zeta potential of APCN, APCN@NA and Tri-NAb. Data of size and zeta potential are presented as means ± s.d. (n = 5 biologically independent samples). Source data are provided as a Source Data file.

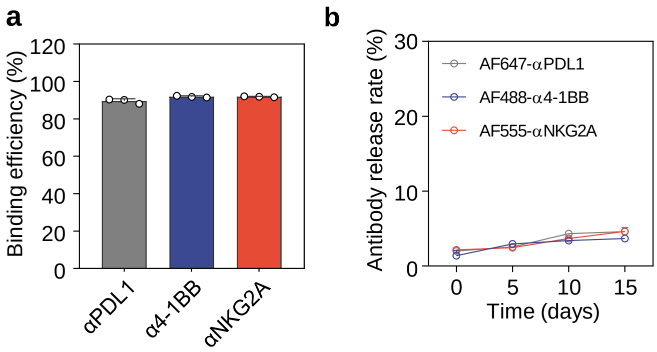

Supplementary Fig. 16. Tri-NAb had good stability in vitro. (a) The binding efficiency of αPDL1, α4-1BB, or αNKG2A. Tri-NAb was prepared by mixing APCN@NA with AF647 labeled αPDL1, AF488 labeled α4-1BB, and AF555 labeled αNKG2A at 4℃ for 24 h. The unbound free mAbs were effectively separated through high-speed centrifugation, and their precise content was quantified using multifunctional microplate reader by detecting three fluorescence intensities. This allowed us to determine the binding efficiency of each mAb to APCN@NA. **(b)** Antibody release rate of αPDL1, α4-1BB, or αNKG2A. Purified Tri-NAb was obtained by high-speed centrifugation and incubated in PBS buffer at 37°C. The released free antibodies were collected by high-speed centrifugation and quantified by fluorescence on days 0, 5, 10, and 15. Data are presented as means ± s.d. (n = 3 biologically independent samples). Source data are provided as a Source Data file.

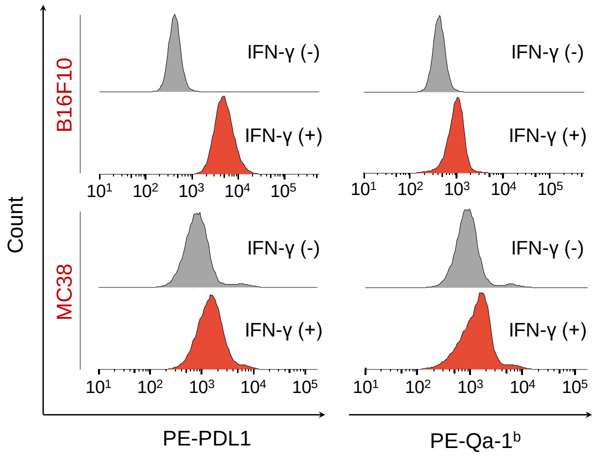

Supplementary Fig. 17. Expression levels of PDL1 and Qa-1^b on stimulated tumor cells. B16-F10 or MC38 cells were stimulated with IFN-γ (20 ng/ml, PeproTech) for 24 h. Flow cytometry detected the expression levels of PDL1 and Qa-1^b. The data were analyzed using FlowJo software (version 10.0.7, TreeStar Inc., Ashland, USA).

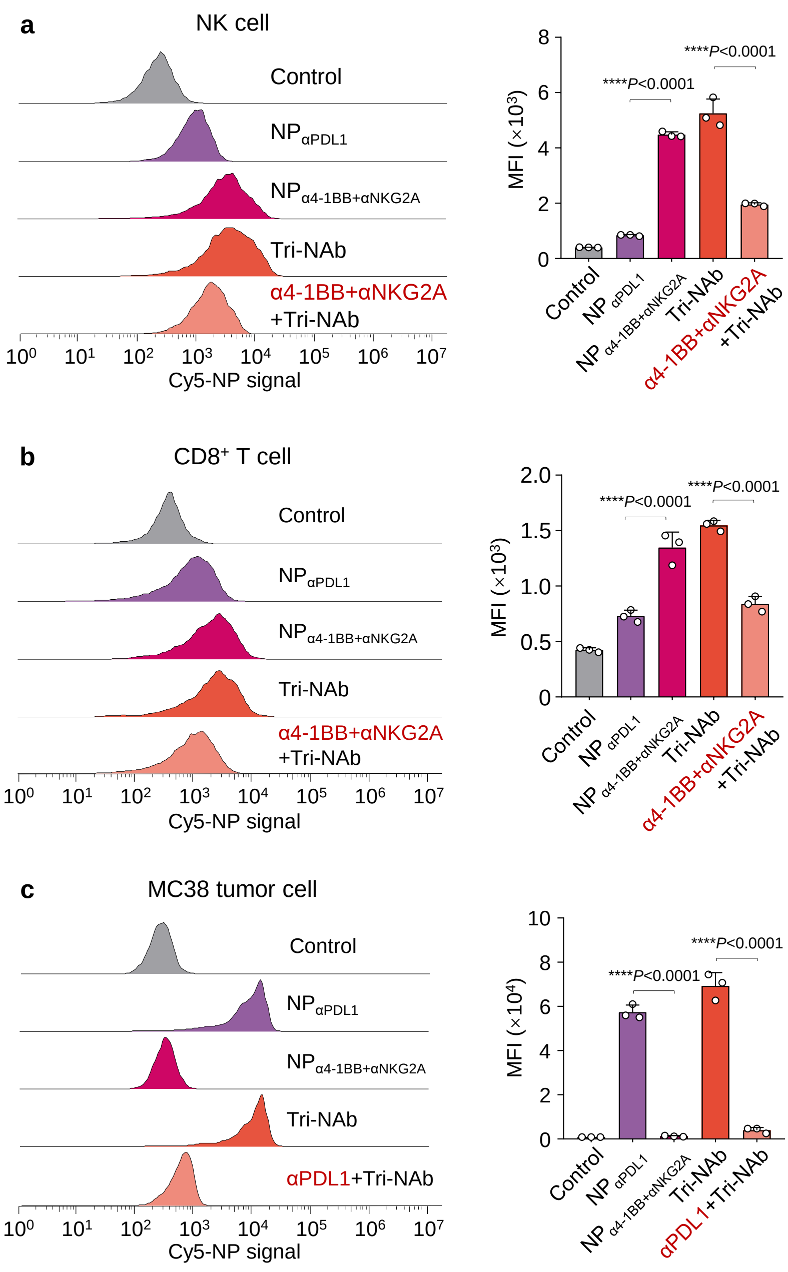

Supplementary Fig. 18. The binding of NPs NK, or CD8^{+} T cells and tumor cells. Representative histogram of the flow cytometry analysis and statistics of NK cells **(a)**, CD8^{+} T cells **(b)**, and MC38 murine colon cancer cells **(c)** after incubation with PBS, Cy5-labeled $NP_{\alpha PDL1}$, $NP_{\alpha 4\text{-}1BB+\alpha NKG2A}$, Tri-NAb, or Tri-NAb with mAbs pretreatment. Data are presented as means ± s.d. (n = 3 biologically independent samples). Statistical significance was calculated by one-way ANOVA with Tukey's post hoc test. ****P < 0.0001. Source data are provided as a Source Data file.

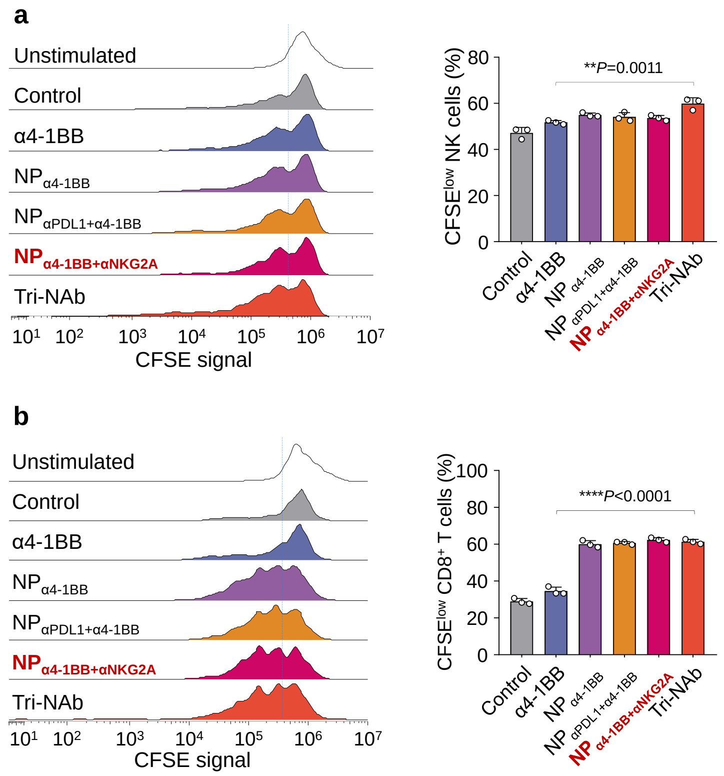

Supplementary Fig. 19. Tri-NAb significantly promoted the proliferation of NK and CD8$^+$ T cells. Representative FACS histograms of CFSE-labeled NK cells **(a)** and CFSE-labeled CD8$^+$ T cells **(b)** after incubation with 1) Control, 2) α4-1BB, 3) $NP_{α4-1BB}$, 4) $NP_{αPDL1+α4-1BB}$, 5) $NP_{α4-1BB+αNKG2A}$, and 6) Tri-NAb. Data are presented as means ± s.d. (n = 3 biologically independent samples). Statistical significance was calculated by one-way ANOVA with Tukey's post hoc test. ***P* < 0.01, *****P* < 0.0001. Source data are provided as a Source Data file.

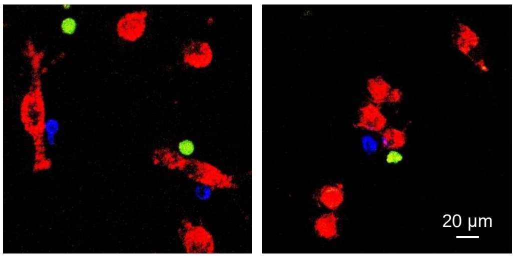

Red: PHK26-MC38 tumor cells Blue: DiD-NK cells
Green: CFSE-CD8$^+$ T cells

Supplementary Fig. 20. **Representative confocal images of NK and CD8$^+$ T cells interacting with MC38 tumor cells after being treated with NP$_{α4\text{-}1BB+αNKG2A}$.**

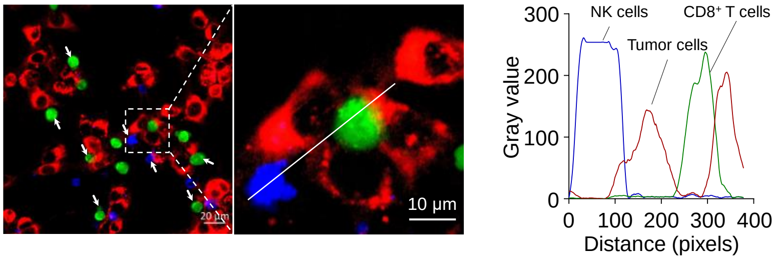

Supplementary Fig. 21. Quantification of intercellular distance. Source data are provided as a Source Data file.

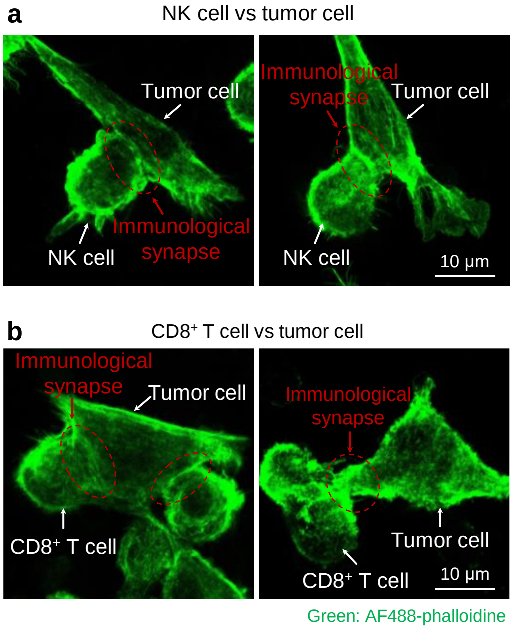

Supplementary Fig. 22. Representative CLSM images showing the formation of immune synapses between NK/T cells and tumor cells. NK **(a)** or $CD8^+$ T cells **(b)** were pretreated with Tri-NAb for activation, and then incubated with the tumor cells, followed by fixation and staining with AF488-phalloidine.

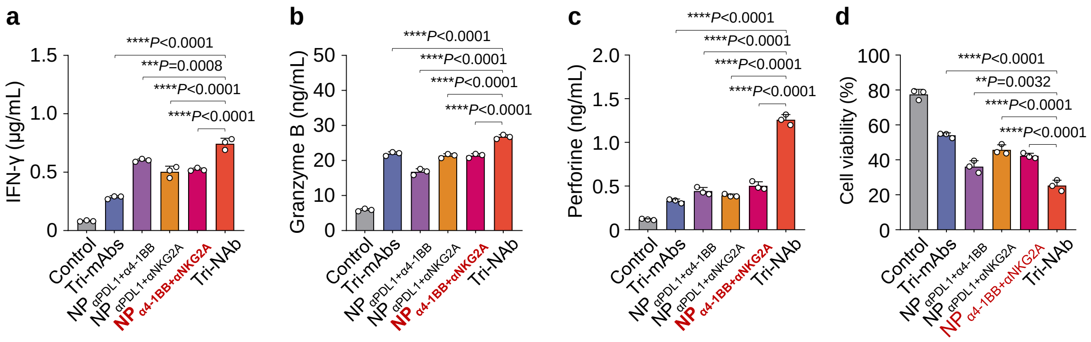

Supplementary Fig. 23. Tri-NAb effectively activated both NK and CD8⁺ T cells in vitro. The release levels of IFN-γ **(a)**, granzyme B **(b)**, and perforin **(c)** in the supernatant of the co-incubation system were examined by ELISA. **(d)** Viability of MC38-luc cells in the co-incubation system. MC38-luc, MC38 cells expressing the luciferase gene. Data are presented as means ± s.d. (n = 3 biologically independent samples). Statistical significance was calculated by one-way ANOVA with Tukey's post hoc test. $^{**}P < 0.01$; $^{***}P < 0.001$; and $^{****}P < 0.0001$. Source data are provided as a Source Data file.

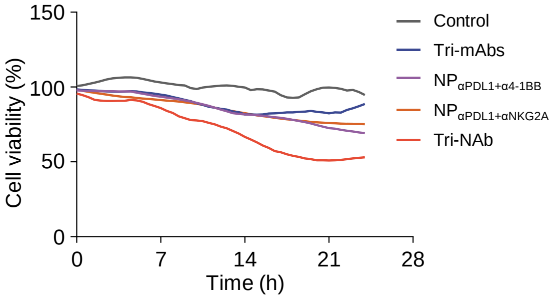

Supplementary Fig. 24. High content analysis (HCA) platform for monitoring the viability of tumor cells. The stimulated PKH26 labelled-MC38 tumor cells (5.0×10^3) were seeded in CellCarrier-96 Ultra Microplates (PerkinElmer) and allowed to attach overnight. The stimulated NK cells (5.0×10^4) and $CD8^+$ T cells (5.0×10^4) were then added. Co-cultured cells were treated with IgG, Tri-mAbs, $NP_{\alpha PDL1+\alpha 4\text{-}1BB}$ $NP_{\alpha PDL1+\alpha NKG2A}$, or Tri-NAb; the concentration of αPDL1, α4-1BB or αNKG2A was 10 µg/mL respectively, and the concentration of IgG was 30 µg/mL. The plates were incubated at 37 °C and 5% CO_2, and cell images were continuously acquired every 30 min using Operetta CLS™ High-Content Analysis System (PerkinElmer) for 24 h. The viability of PKH26 labelled-MC38 tumor cells was evaluated using Harmony® high-content analysis software based on cellular phenotypes and fluorescence distribution parameters. Source data are provided as a Source Data file.

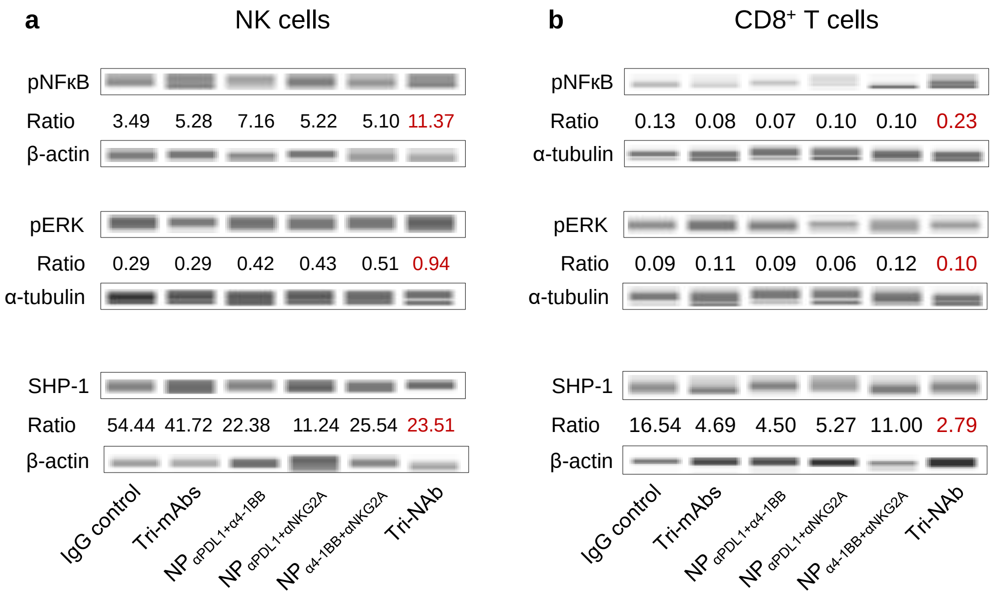

Supplementary Fig. 25. Levels of SHP-1, ERK/NFκB phosphorylation of NK and CD8$^+$ T cells after PDL1$^+$ Qa-1b$^+$ tumor cells exposure. The stimulated NK or CD8$^+$ T cells were co-incubated with IFN-γ-stimulated MC38 tumor cells, and then were treated with IgG control, Tri-mAbs, $NP_{αPDL1+α4\text{-}1BB}$, $NP_{αPDL1+αNKG2A}$, $NP_{α4\text{-}1BB+αNKG2A}$, or Tri-NAb, where the concentration of αPDL1, α4-1BB, or αNKG2A was 10 μg/mL respectively, and the concentration of mAbs was 30 μg/mL. After co-incubation for 24 h, NK or CD8$^+$ T cells were collected and subjected to western blot using Jess Automated Western Blot System (ProteinSimple, Bio-Techne, USA). Data were analyzed using Compass for SW software (version 6.3.0, ProteinSimple, USA).Phospho-NF-kappaB p65(Ser536) Rabbit mAb (Product code: 3033, Cell Signaling Technology, 1:50 dilution), Phospho-p44/42 MARK(Erk1/2) (Thr202/Tyr204) XP Rabbit mAb (Product code: 4370, Cell Signaling Technology, 1:100 dilution), PTPN6 (SHP-1) Polyclonal antibody (Product code: 24546-1-APR, Proteintech, 1:250 dilution), Alpha Tubulin Polyclonal antibody (Product code: 11224-1-AP, Proteintech, 1:250 dilution), and Beta Actin Recombinant antibody (Product code: 81115-1-RR, Proteintech, 1:200 dilution) were used and then signals were detected by HRP-conjugated secondary goat anti-rabbit (Product code: 042-206, ProteinSimple, 1:200 dilution). Source data are provided as a Source Data file.

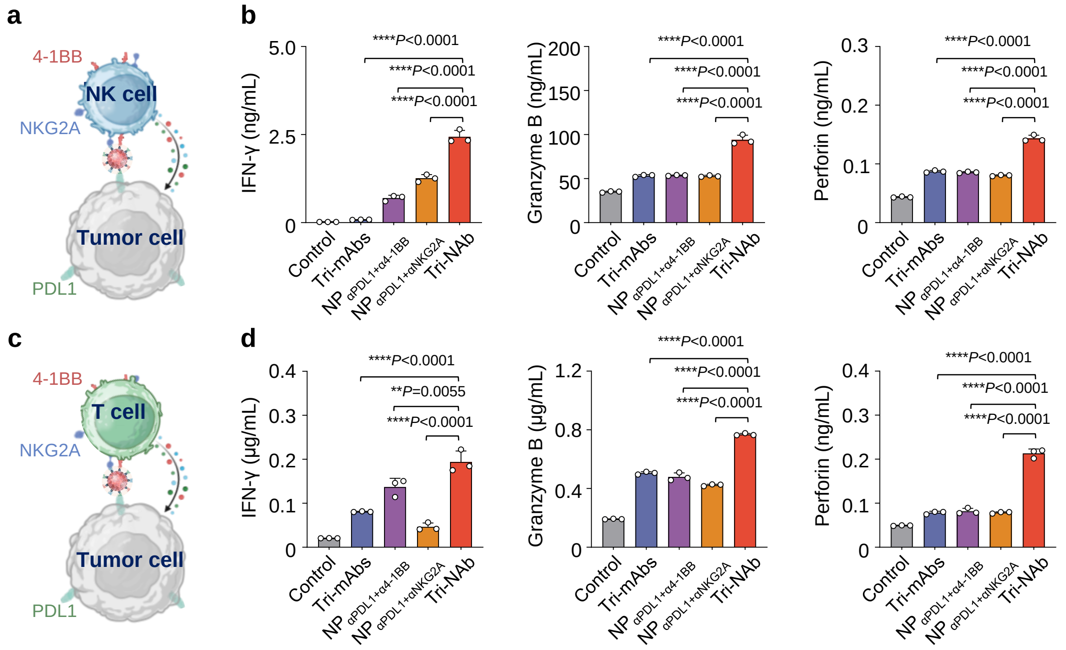

Supplementary Fig. 26. Tri-NAb improved the levels of IFN-γ, perforin, and granzyme B released by NK or CD8$^+$ T cells respectively in vitro. Schematic representation of the killing effect exerted by NK (**a**) or CD8$^+$ T cells (**c**). The release level of IFN-γ, Granzyme B and Perforin detected by ELISA during NK (**b**) or CD8$^+$ T cells (**d**) co-incubation with tumor cells. The stimulated B16-F10 cells (5.0×10^3) were seeded in 96-well plates (Corning) and allowed to attach overnight. The stimulated NK cells (5.0×10^4) or CD8$^+$ T cells (5.0×10^4) were then added. Co-cultured cells were treated with IgG control, Tri-mAbs, NP$_{\alpha PDL1+\alpha NKG2A}$, NP$_{\alpha PDL1+\alpha 4\text{-}1BB}$, or Tri-NAb; the concentration of αPDL1, α4-1BB or αNKG2A was 20 μg/mL respectively, and the concentration of IgG was 60 μg/mL. After co-incubation for 24 h, the supernatant was collected, and the cytokines in the supernatant were quantified via mouse IFN-γ ELISA Kit (Dakewe Biotech), mouse Granzyme B ELISA Kit (Abcam) and mouse Perforin 1 ELISA Kit (Abbexa) according to the manufacturer's protocol. Data are presented as means ± s.d. (n = 3 biologically independent samples). Statistical significance was calculated by one-way ANOVA with Tukey's post hoc test. $**P < 0.01$; $****P < 0.0001$. Supplementary Fig. 26a and 26c were created with BioRender.com under a CC-BY-NC-ND license. Source data are provided as a Source Data file.

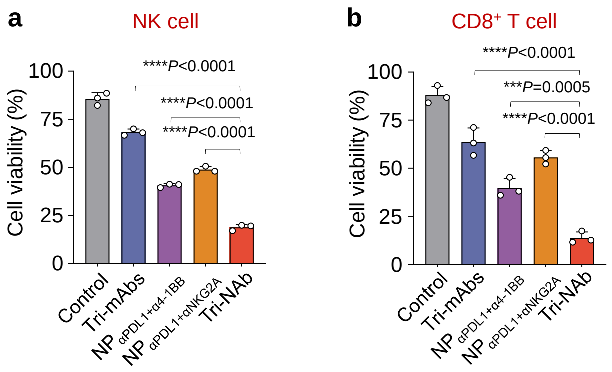

Supplementary Fig. 27. Tri-NAb enhanced the anti-tumor killing effect of NK or CD8⁺ T cells in vitro. MC38-luc cells (5.0×10^3) were seeded in 96-well plates (Costar) and stimulated with IFN-γ (20 ng/ml, PeproTech) for 24 h. The stimulated NK (5.0×10^4) or $CD8^+$ T cells (5.0×10^4/well) were added 12 h later. Co-cultured cells were treated with IgG, Tri-mAbs, $NP_{\alpha PDL1+\alpha 4\text{-}1BB}$, $NP_{\alpha PDL1+\alpha NKG2A}$ or Tri-NAb; the concentration of αPDL1, α4-1BB, or αNKG2A was 20 µg/mL respectively, and the concentration of IgG was 60 µg/mL. After co-incubation for 48 h, the cell culture supernatant was discarded and 100 µL D-Luciferin potassium salt solution (150 µg/mL) was added to the reaction for 5 minutes to enter the cells. Luminescence intensity analysis was performed immediately with the Infinite® 200 PRO microplate reader (Tecan). Viability of MC38-luc cells in the NK (**a**) or $CD8^+$ T cells (**b**) incubation system. Data are presented as means ± s.d. (n = 3 biologically independent samples). Statistical significance was calculated by one-way ANOVA with Tukey's post hoc test. ***$P < 0.001$; ****$P < 0.0001$. Source data are provided as a Source Data file.

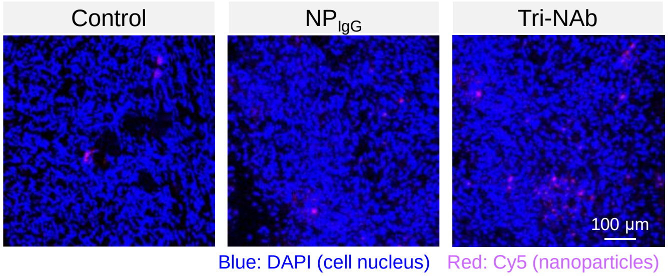

Supplementary Fig. 28. Representative fluorescent images of tumor tissues. Subcutaneous MC38 tumor model was established in female C57BL/6 mice and Cy5-labelled NP_{IgG} or Cy5-labelled Tri-NAb was administrated every three days for three repeats post tumor inoculation, the injection dose of αPDL1, α4-1BB, and αNKG2A was 2.5 mg/kg. Tumor tissues were harvested. Frozen sections were made, stained with DAPI (blue) and observed with CLSM.

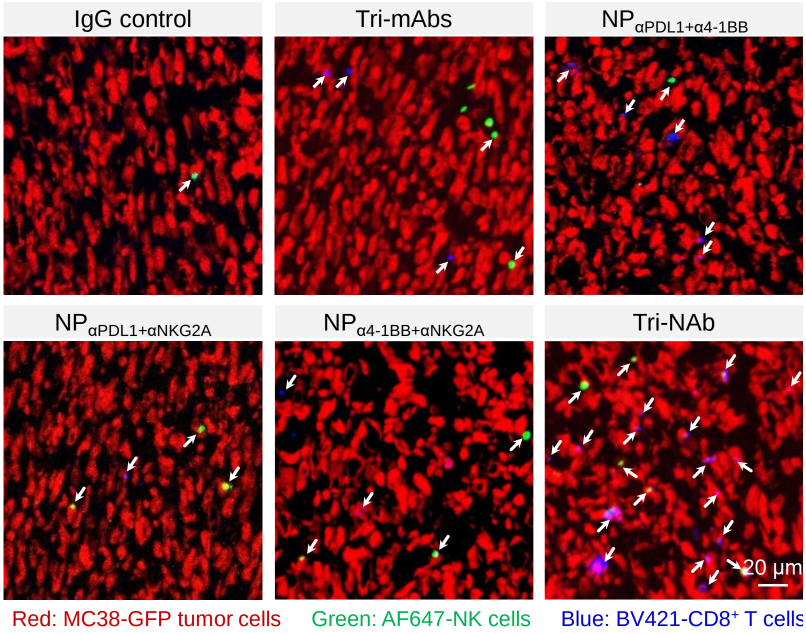

Supplementary Fig. 29. Representative confocal images of NK and CD8$^+$ T cells interacting with MC38 tumor cells in vivo. Male C57BL/6 mice bearing MC38 colon cancer were treated with corresponding formulations on a schedule of an intravenous injection every three days to a total of three injections (q3dx3), receiving IgG, Tri-mAbs, $NP_{\alpha PDL1+\alpha 4\text{-}1BB}$, $NP_{\alpha PDL1+\alpha NKG2A}$, $NP_{\alpha 4\text{-}1BB+\alpha NKG2A}$, or Tri-NAb (the doses of αPDL1, α4-1BB or αNKG2A: 2.5 mg/kg respectively). The tumor tissues were harvested 24 h post-last injection, sectioned into 10 μm pieces, and stained with anti-CD8 and anti-NK1.1 antibodies for CLSM observation.

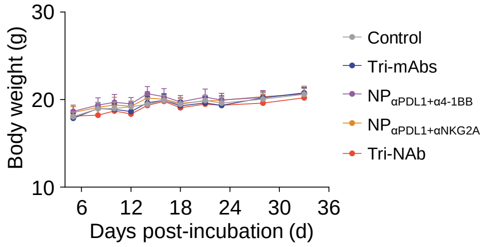

Supplementary Fig. 30. Body weight changes in female C57BL/6 mice bearing MC38 tumors throughout the course of the experiment. Data are presented as means ± s.d. (n = 10 mice per group). Source data are provided as a Source Data file.

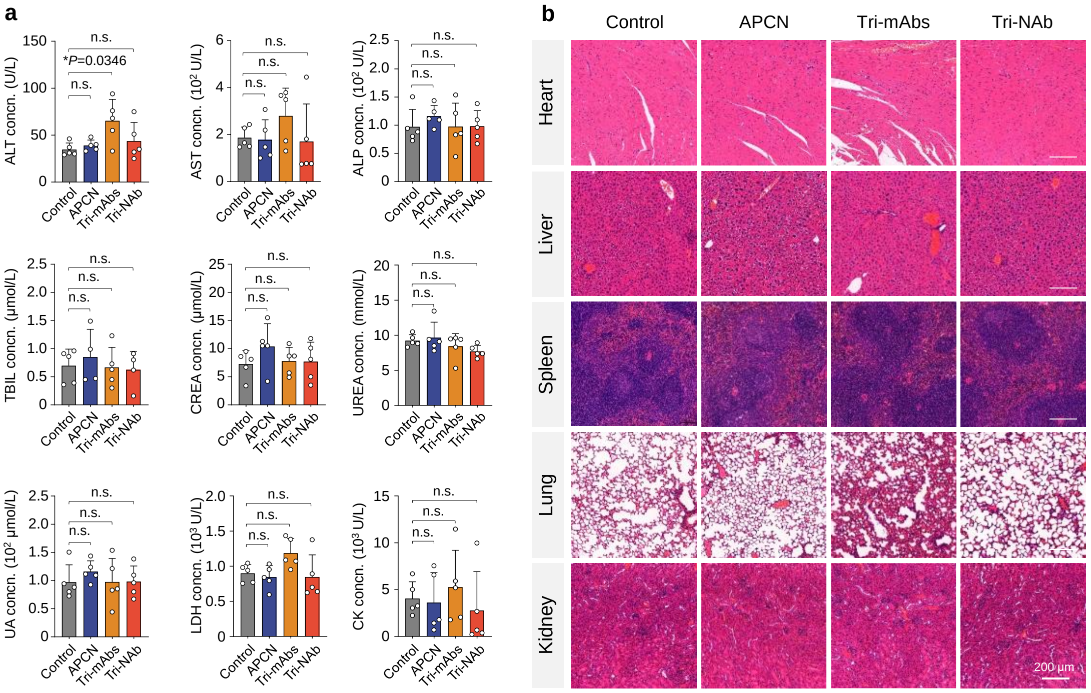

Supplementary Fig. 31. In vivo biological safety evaluation of Tri-NAb. Female ICR mice (6-8 weeks) were intravenously injected with PBS, APCN, Tri-mAbs or Tri-NAb (αPDL1, α4-1BB or αNKG2A: 2.5 mg/kg, where the dose of each mAb was the same as that used in the efficacy study for effective detection) every three days for three repeats. One-month post-last injection, peripheral blood and main organs including the heart, liver, spleen, lung, and kidney, were collected for serum biochemical and histological analyses. **(a)** Serum biochemical analysis for evaluating liver, renal and cardiac functions. Alanine aminotransferase (ALT), aspartate aminotransferase (AST), alkaline phosphatase (ALP), total bilirubin (TBIL), creatinine (CREA), urea (UREA), uric acid (UA), lactate dehydrogenase (LDH), and creatine kinase (CK) were examined by Automatic Aralyzer (HITACHI). n.s., not significant. Data are presented as means ± s.d. (n = 5 biological independent mice). **(b).** Representative hematoxylin & eosin (H&E) staining images of main organs by a digital pathology scanning system (P250 FLASH, 3D Histech). Statistical significance was calculated by one-way ANOVA with Tukey's post hoc test. *$P < 0.05$. Source data are provided as a Source Data file.

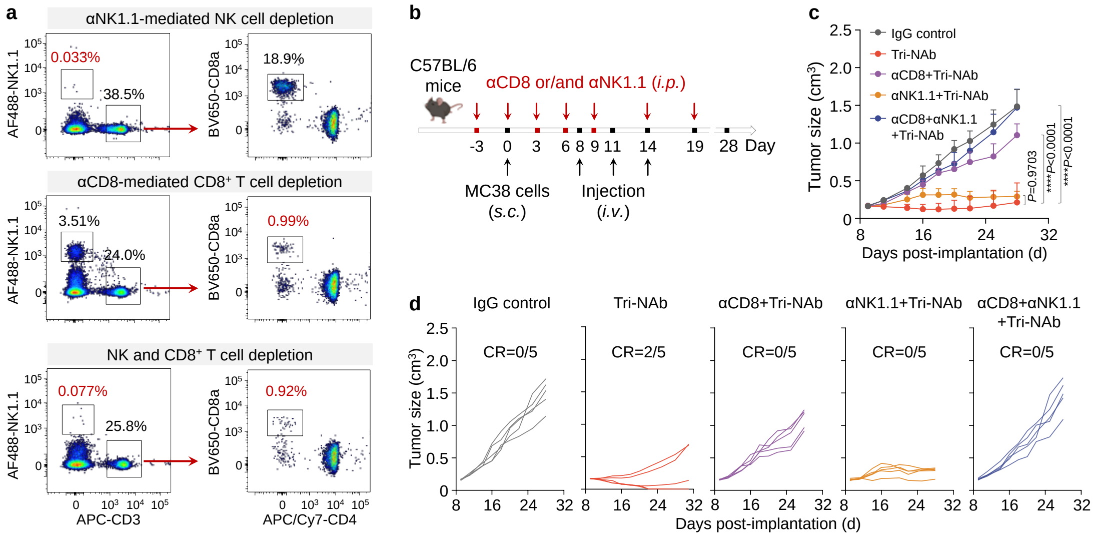

Supplementary Fig. 32. CD8⁺ T cells played a dominant role in Tri-NAb-mediated tumor growth suppression. (**a**) Representative scatter plots of flow cytometry showing the proportion of NK/CD8⁺ T cells in the peripheral blood of mice after antibody-mediated depletion. (**b**) A subcutaneous MC38 tumor model was established in male C57BL/6 mice. According to the experimental protocol, NK and CD8⁺ T cells were depleted via intraperitoneal injection of 150 μg anti-NK1.1 mAb or/and 125 μg anti-CD8 mAb on specific days. Tri-NAb was administrated every three days for three repeats since the 8th day post-tumor inoculation; the injection dose of αPDL1, α4-1BB, and αNKG2A was 2.5 mg/kg. Average (**c**) and individual (**d**) tumor growth curves of MC38 tumors after being treated with Tri-NAb with or without NK or/and CD8⁺ T cells depletion. CR, complete remission rate. The statistical data are presented as the means ± s.d. (n = 5 biological independent mice). Statistical significance was calculated by one-way ANOVA with Tukey's post hoc test. ****$P < 0.0001$. Supplementary Fig. 32b was created with BioRender.com under a CC-BY-NC-ND license. Source data are provided as a Source Data file.

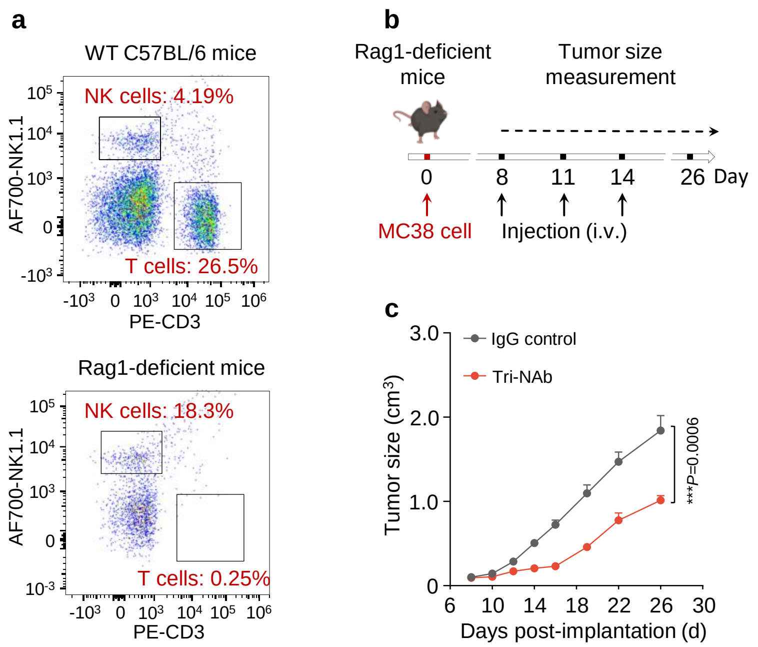

Supplementary Fig. 33. Tri-NAb mildly controlled tumor progression in Rag1-deficient mice. (**a**) Representative scatter plots of flow cytometry showing the proportion of NK and T cells in peripheral blood of immunocompetent female C57BL/6 mice and female Rag1-deficient mice. (**b**) Experimental scheme of subcutaneous MC38 murine colon cancer model in Rag1-deficient mice. Tri-NAb with equivalent doses of αPDL1, α4-1BB, and αNKG2A (2.5 mg/kg each) was intravenously (*i.v.*) administered on days 8, 11, and 14 post MC38 tumor inoculation. (c) Tumor growth curves of MC38 tumors. The statistical data are presented as the means ± s.d. (n = 5 biological independent mice). Statistical significance was calculated by paired t-test with two-tailed. ***$P < 0.001$. Supplementary Fig. 33b was created with BioRender.com under a CC-BY-NC-ND license. Source data are provided as a Source Data file.

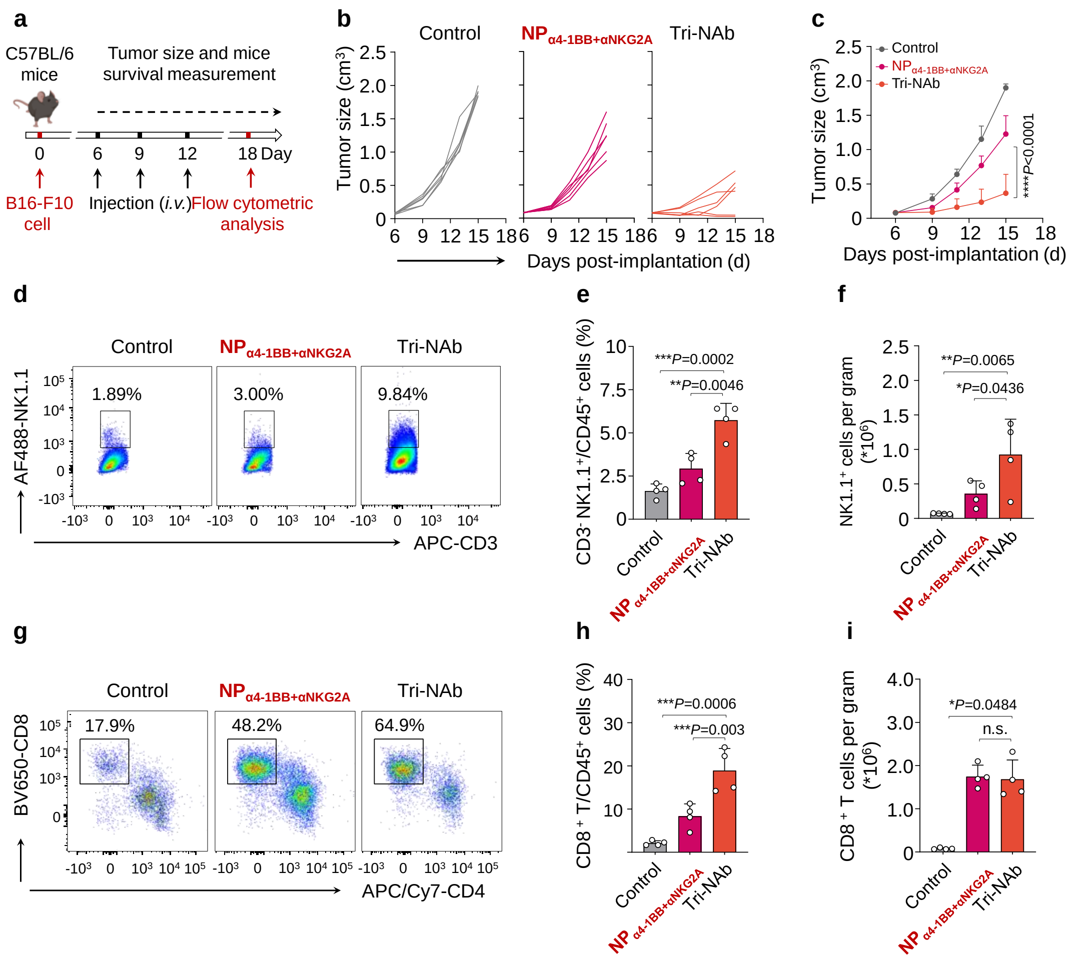

Supplementary Fig. 34. Tri-NAb effectively inhibited the progression of murine melanoma. (**a**) Experimental scheme of subcutaneous B16-F10 (4.0×10^5) murine melanoma model in male C57BL/6 mice. Different formulations with equivalent doses of αPDL1, α4-1BB, and αNKG2A (2.5 mg/kg each) were intravenously (*i.v.*) administered via the tail on days 6, 9, and 12 after B16-F10 tumor inoculation. Individual (**b**) and average (**c**) tumor growth kinetics in different formulations. Growth curves represent means ± s.d. (n = 6 mice per group). Representative scatter plots of flow cytometry showing the number of NK cells as a percentage of $CD3^-$ cell population (**d**) or $CD8^+$ T cells as a percentage of $CD3^+$ cell population (**g**) in the tumor after different treatments as indicated. Quantitative results of the number of NK (**e**) or $CD8^+$ T cells (**h**) as a percentage of the total $CD45^+$ cell population in the tumor. Quantitative results of the total number of NK (**f**) or $CD8^+$ T cells (**i**) per gram of tumor tissue. FACS data are presented as means ± s.d. (n = 4 biological independent mice). Statistical significance was calculated by one-way ANOVA with Tukey's post hoc test. **P* < 0.05; ***P* < 0.01; ****P* < 0.001. Supplementary Fig. 34a was created with BioRender.com under a CC-BY-NC-ND license. Source data are provided as a Source Data file.

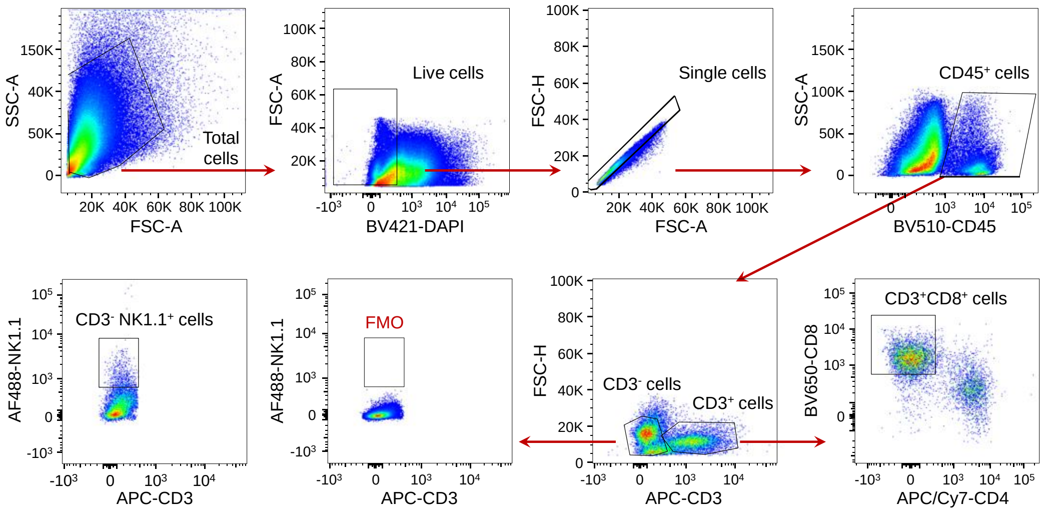

Supplementary Fig. 35. Gating strategy for tumor infiltrating lymphocyte. Immune cell population was gated based on the expression of CD45, CD45$^+$ cells were further gated to identify CD3$^+$ and CD3$^-$ cells. The tumor-infiltrating NK cell population identified as CD45$^+$CD3$^-$NK1.1$^+$, and FMO control for anti-NK1.1 was also included in flow cytometric analysis. Additionally, CD45$^+$CD3$^+$ cells were gated to analyze tumor-infiltrating CD8$^+$ and CD4$^+$ T cells. Cell populations were gated sequentially following arrows.

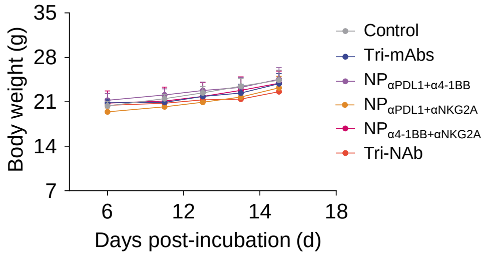

Supplementary Fig. 36. Body weight changes in male C57BL/6 mice bearing B16-F10 tumors throughout the course of the experiment. Data are presented as means ± s.d. (n = 6 mice per group). Source data are provided as a Source Data file.

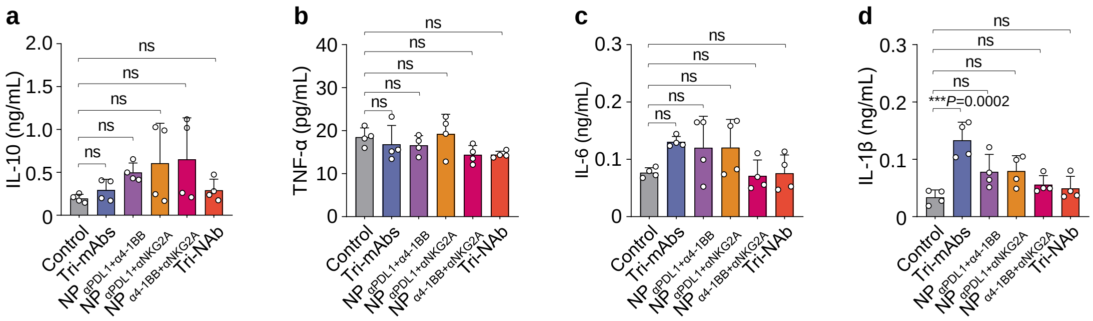

Supplementary Fig. 37. The levels of serum inflammatory cytokines in the peripheral blood. The levels of IL-10 **(a)**, TNF-α **(b)**, IL-6 **(c)**, and IL-1β **(d)** in the peripheral blood of the treated mice were examined by ELISA. n.s., not significant. Data are presented as means ± s.d. (n = 4 biological independent mice). Statistical significance was calculated by one-way ANOVA with Tukey's post hoc test. ***$P < 0.001$. Source data are provided as a Source Data file.

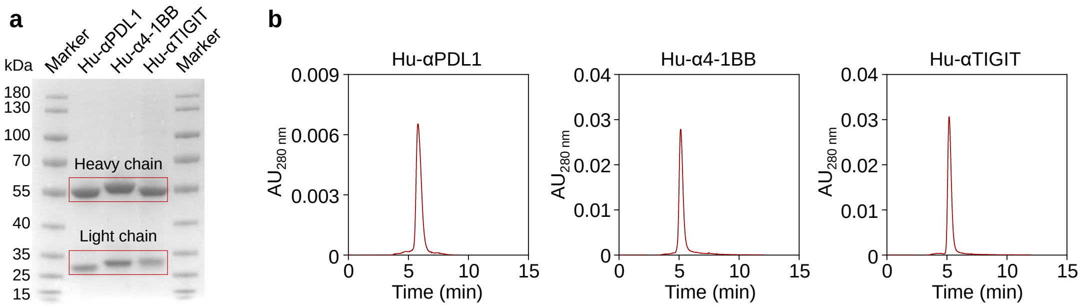

Supplementary Fig. 38. Characterization of humanized monoclonal antibodies. (a) A representative reducing SDS-PAGE gel showing the light and the heavy chains of anti-human PDL1 antibody (Hu-αPDL1), anti-human 4-1BB antibody (Hu-α4-1BB), and anti-human TIGIT antibody (Hu-αTIGIT). **(b)** Representative chromatograms of Hu-αPDL1, Hu-α4-1BB, and Hu-αTIGIT demonstrate antibody purity exceeding 95.0% as determined by HPLC analysis (Waters, XBridge Protein BEH SEC Column, 200Å). Source data are provided as a Source Data file.

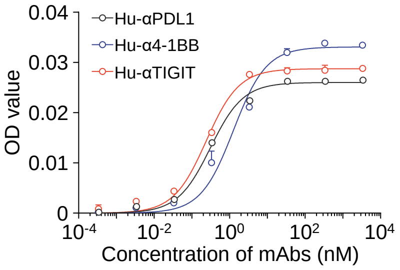

Antibody	Kd value/M
Hu-αPDL1	3.11×10^{-10}
Hu-αTIGIT	2.41×10^{-10}
Hu-α4-1BB	1.28×10^{-9}

Supplementary Fig. 39. The affinity of humanized monoclonal antibodies against the corresponding antigens. Kd represents the equilibrium dissociation constant. Data are presented as means ± s.d. (n = 3 biologically independent samples). Source data are provided as a Source Data file.

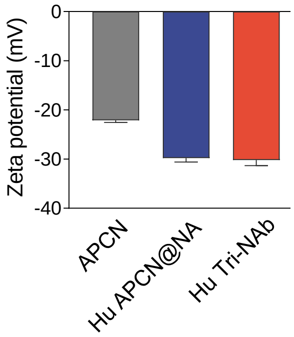

Supplementary Fig. 40. The zeta potential of Hu APCN@NA and Hu Tri-NAb. Data are presented as means ± s.d. (n = 5 biologically independent samples). Source data are provided as a Source Data file.
