# Supplementary material for: Orchestrating NK and T cells via tri-specific nano-antibodies for synergistic antitumor immunity
Source: Nat Commun. 2024 Jul 23;15:6211. doi: 10.1038/s41467-024-50474-y (PMC11266419; doi:10.1038/s41467-024-50474-y)
Supplement: Supplementary file 3 — Description of Additional Supplementary Files [file 41467_2024_50474_MOESM3_ESM.pdf]

Description of Additional Supplementary Files

Supplementary Video 1. NK cells treated with Tri-NAb did not harm each other. CFSE-labeled NK cells (green) were treated with Tri-NAb (10 μg/mL per mAb), and cell behavior was evaluated within 6 h using the ImageXpress® Micro Confocal High-Content Imaging System (Molecular Devices) to observe whether NK cells were harmed. Before imaging, propidium iodide dye (50 μg/mL) was added to distinguish between living and dead cells (red).

Supplementary Video 2. $CD8^+$ T cells treated with Tri-NAb did not harm each other. CFSE-labeled $CD8^+$ T cells (green) were treated with Tri-NAb (10 μg/mL per mAb), and cell behavior was evaluated within 6 h using the ImageXpress® Micro Confocal High-Content Imaging System (Molecular Devices) to observe whether $CD8^+$ T cells were harmed. Before imaging, propidium iodide dye (50 μg/mL) was added to distinguish between living and dead cells (red).

Supplementary Video 3. The fratricide in NK and $CD8^+$ T cells did not occur after treatment with Tri-NAb. Co-incubated DiD-labeled NK (blue) and CFSE-labeled $CD8^+$ T cells (green) were treated with Tri-NAb (10 μg/mL per mAb), and cell behavior was evaluated within 6 h using the ImageXpress® Micro Confocal High-Content Imaging System (Molecular Devices) to observe whether binding led to effector cell fratricide. Before imaging, propidium iodide dye (50 μg/mL) was added to distinguish between living and dead cells (red).
